# Case report: Open bite as an extrapyramidal side effect with aripiprazole, a dopamine partial agonist

**DOI:** 10.3389/fpsyt.2022.976387

**Published:** 2022-09-06

**Authors:** Satoko Sumi, Takahiko Nagamine, Koji Sumi, Reona Aijima, Kyoko Oka, Akira Toyofuku

**Affiliations:** ^1^Section of Pediatric Dentistry, Department of Oral Growth and Development, Fukuoka Dental College, Fukuoka, Japan; ^2^Sunlight Brain Research Center, Hofu, Japan; ^3^Department of Psychosomatic Dentistry, Graduate School of Medical and Dental Sciences, Tokyo Medical and Dental University, Tokyo, Japan; ^4^Sumi Orthodontic Clinic, Saga, Japan; ^5^Department of Oral and Maxillofacial Surgery, Faculty of Medicine, Saga University, Saga, Japan

**Keywords:** open bite, extrapyramidal side effect, aripiprazole, orthodontic treatment, adolescent

## Abstract

Long-term, fixed-point posttreatment observation of orthodontically treated patients provided us with the opportunity to capture the onset, development, and improvement of open bite, a type of malocclusion. Based on the chronological sequence of events, i.e., a tendency for open bite to worsen with increasing aripiprazole dosage and to improve with decreasing dosage, it was inferred that the onset of malocclusion was caused by extrapyramidal symptoms related to aripiprazole dosage. Physicians should be aware of this side effect when prescribing aripiprazole to children and adolescents. Careful consideration of medication history is necessary when dentists treat open bite in children and adolescents.

## Introduction

**Figure 1:**
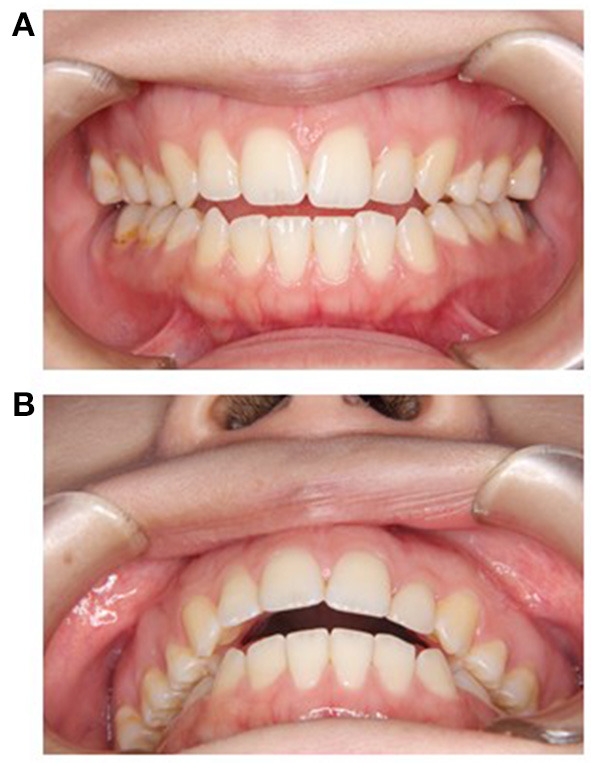
Open bite: **(A)** front view, **(B)** occlusal view.

Open bite, as shown in Figure 1, can be caused by oral habits such as thumb sucking and tongue thrusting, or by overgrowth of the mandible during growth (1). However, open bites that cannot be explained by the above causes are observed in clinical practice and may be due to drug-induced side effects. We experienced a case of open bite as an extrapyramidal side effect (EPS) caused by aripiprazole. Aripiprazole is classified as an atypical antipsychotic, and its pharmacological action is that of a dopamine partial agonist, with few EPS (2). This is the first report of aripiprazole-induced open bite. In cases of open bite of unknown etiology, close attention should be paid to the patient's medication history.

## Case report

**Figure 2:**
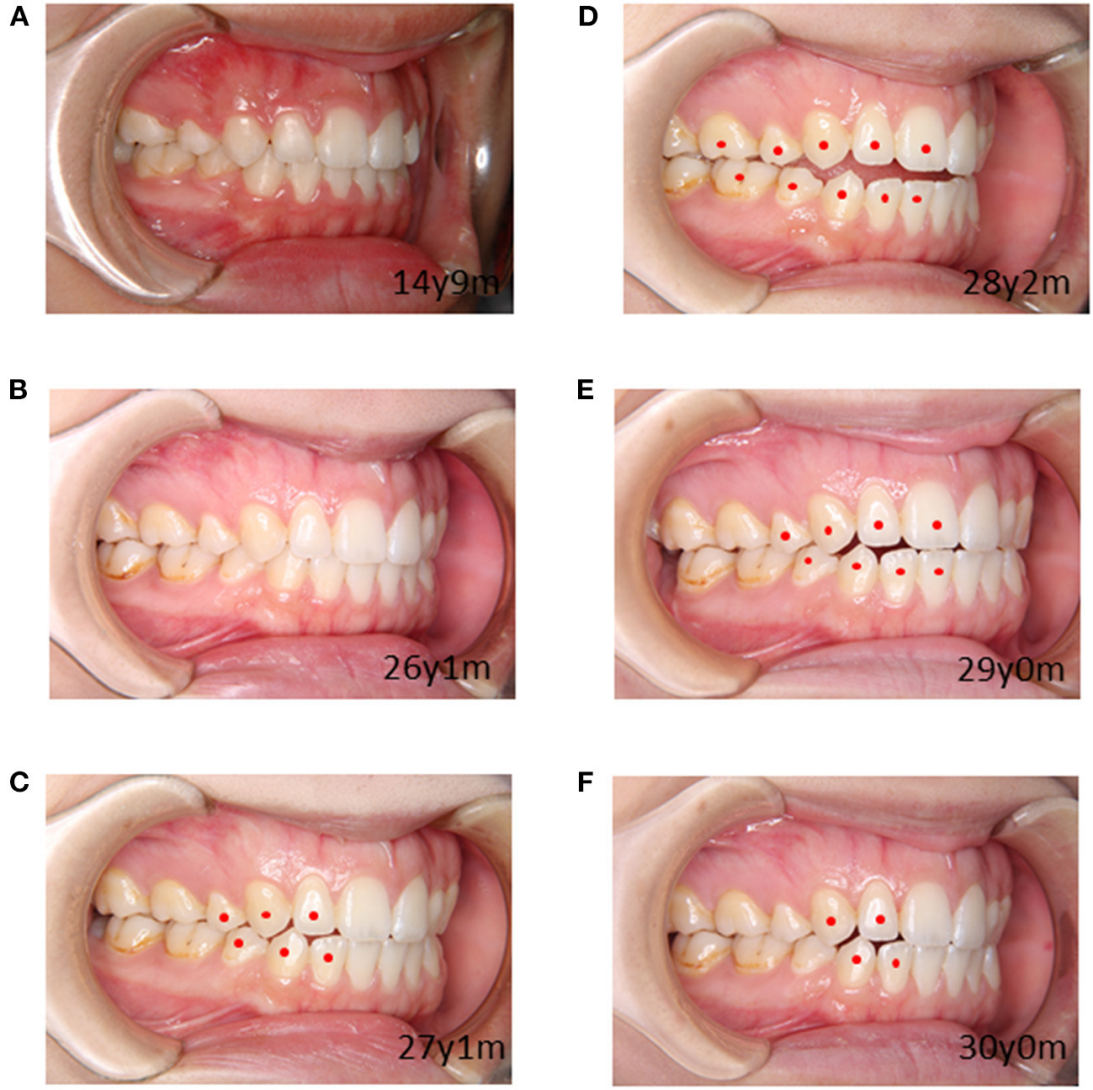
Changing images with age and degrees of open bites. Circle symbols are placed on non-contacting teeth, and the number of separated tooth type indicate the degree of open bite: **(A)** Degree 0, **(B)** Degree 0, **(C)** Degree 3, **(D)** Degree 5, **(E)** Degree 4, **(F)** Degree 2.

**Figure 3:**
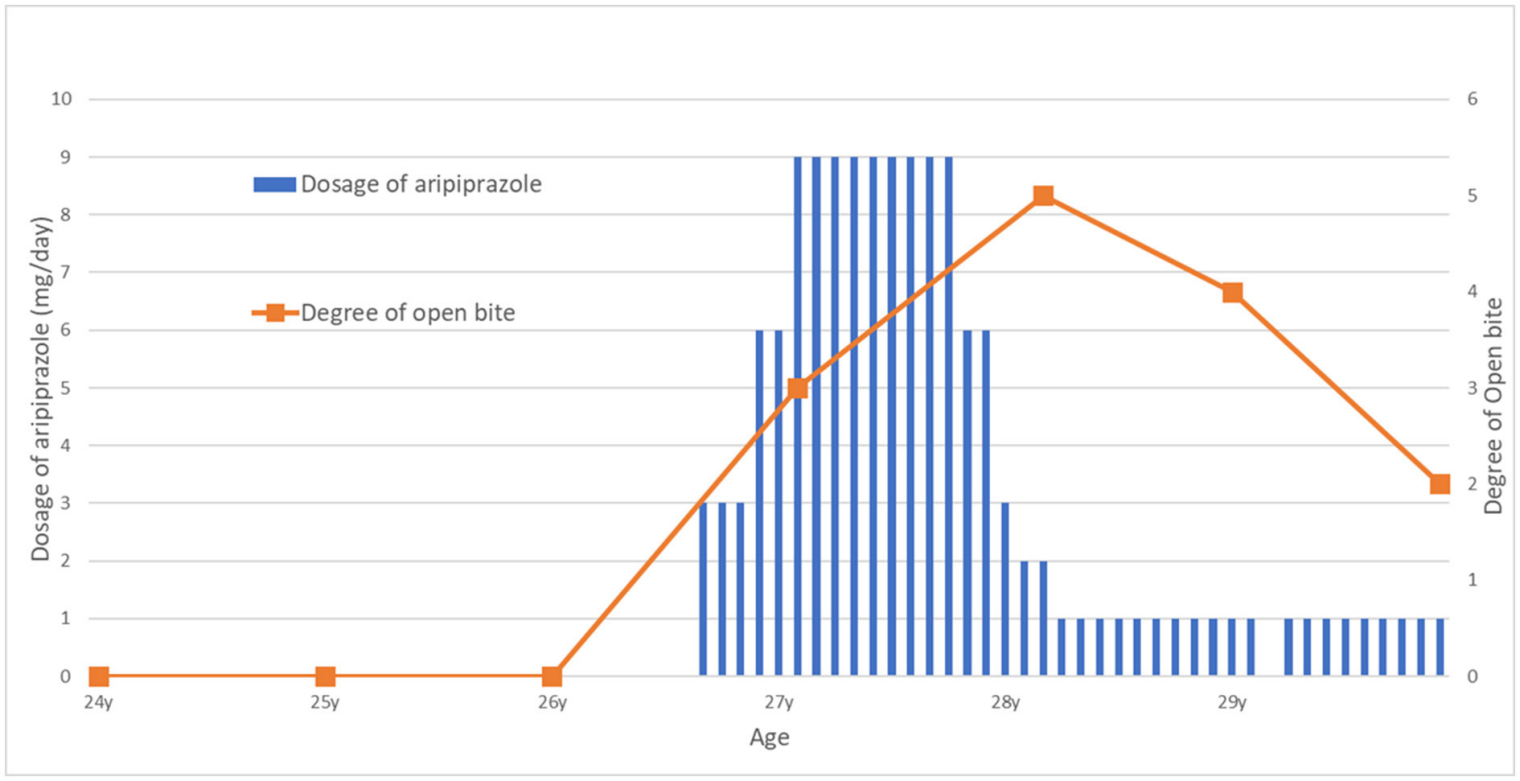
Relationship between aripiprazole dosage and degree of open bite.

The patient was a female with malocclusion who had orthodontic treatment at the age of 12 years old. Two years of active treatment was followed by 3 years of retention using a removable retentive appliance. After the completion of the retention period, the

patient was observed annually, and her dentition and occlusion were in good condition. She developed depression at the age of 24 and received medication at a psychiatric clinic. Various selective serotonin reuptake inhibitors were tried for 3 years with limited effect, all discontinued due to gastrointestinal symptoms, and dental examinations during this time showed no evidence of open bite. However, at the observation at the age of 27 years and 1 month, more than 12 years after the end of active treatment, open bite was observed. At this time, the patient was treated with aripiprazole for depression. Aripiprazole was started at 3 mg/day and increased to 6 mg/day after 3 months, with mild improvement in depression. Since the cause could not be identified, active orthodontic treatment was avoided and follow-up observation was continued, but the open bite worsened further. The degree of open bite was determined using the number of separated teeth as an indicator (Figure 2). After another 3 months, the dosage was increased to 9 mg/day for 8 months, at which time the open bite became more severe. The dose was subsequently reduced to 6, 3, and 1 mg/day, but the depression did not worsen and the open bite gradually improved (Figure 3). Dental care and observation were discontinued due to the patient's relocation. However, when the patient was contacted by phone, it was confirmed that she is not currently depressed, is not taking aripiprazole or any other medication, and has no bite problems. During the course of this case, there was no evidence of dystonia, dyskinesia, or other involuntary movements.

For ethical considerations, we have written the article in such a way that the patient cannot be identified, and we have obtained written consent from the patient for the publication of this article.

## Discussion

Abnormal movements in the orofacial region include dystonia, dyskinesia, lip biting, tongue thrusting habit, and open bite. Most of these are unexplained, but drug-induced movement disorders such as EPS should not be overlooked, as improvement can be expected with discontinuation of medication. Antipsychotic-naive individuals, elderly, and those with a history of central nervous system disorders or alcohol/substance abuse are at higher risk of developing EPS (3). Among these risk factors, the first administration of antipsychotic medication was applicable in this case.

Open bite is a type of malocclusion in which the upper and lower teeth do not touch when the mouth is completely closed, leaving a gap between the upper and lower teeth. The best and most common treatment for an open bite is an orthodontic approach. Braces can help balance a bite by pushing and pulling teeth into their proper position. In severe cases or in situations where all other orthodontic treatments have failed, some patients resort to orthognathic surgery of the jaws. In case of untreated, open bite can have effects such as lisping and other speech difficulties, difficulty in eating certain foods such as pasta, increased wear on the back teeth, and cause physical complaints such as stiff shoulders and headaches. There are four main causes of open bite: thumb or pacifier sucking, tongue protrusion, temporomandibular joint disorder, and skeletal problems (1). However, there are cases of unexplained malocclusion that do not have these causes. Since occlusion is achieved by the movement of the mandible in conjunction with the masseter, medial pterygoid, and temporalis muscles, open bite can also be caused by muscle tension. Thus, agents that affect muscle tone may induce an open bite. Although the patient was taking more than 20 different medications, the open bite developed in association with the timing of aripiprazole medication and tended to worsen with increasing aripiprazole dosage and improve with decreasing dosage, with a Naranjo side effect scale of 5. This scale includes all of the usual characteristics that are important in assessing causality. Total scores range from −4 to 13, with a score of 5 or higher being considered probable (4). In addition, there was no history of strong D2 receptor blockers such as risperidone in her medication history. Therefore, the open bite in this case is very likely caused by aripiprazole. Antipsychotic-induced open bite was reported by Nakamura with haloperidol, a typical antipsychotic (5). In the case reported by Nakamura, the patient had generalized EPS symptoms, and open bite was one of the symptoms of EPS. However, our case differs from this report in that there was no obvious EPS symptom other than open bite. This may be because aripiprazole is an atypical antipsychotic and is associated with a lower incidence of systemic EPS than first-generation antipsychotics such as haloperidol.

All currently marketed antipsychotic drugs, including aripiprazole, have dopamine D2 receptor blocking properties, and all are at risk of inducing EPS to varying degrees. EPS, also called drug-induced movement disorders, is one of the most common adverse drug effects experienced by patients taking antipsychotic medications. It was first described in 1952 after chlorpromazine-induced symptoms resembling Parkinson disease (6). A broad spectrum of EPS symptoms exists, ranging from acute symptoms such as acute dystonia, akathisia, and parkinsonism to chronic forms such as tardive akathisia and tardive dyskinesia. The mechanism that elicits EPS is mainly the blockade of dopamine D2 receptors in the nigrostriatal pathway, but dopamine D2 blockade in the caudate nucleus and other basal ganglia is also believed to contribute significantly to the development of EPS (7). Centrally-acting, dopamine-receptor blocking agents, namely the first-generation antipsychotics including haloperidol and phenothiazine neuroleptics, are the most common medications associated with EPS. While EPS occurs less frequently with atypical antipsychotics, the risk of EPS increases with dose escalation (8). A possible method to alleviate EPS is to block excessive transmission of nigrostriatal acetylcholine, such as benztropine, but it was not used in this case because of concerns about the emergence of new adverse effects on gastrointestinal motility and cognitive function.

Aripiprazole is an atypical antipsychotic used to treat schizophrenia, depression, bipolar disorder, and obsessive-compulsive disorder, but unlike other antipsychotic drugs, its pharmacological action is that of a dopamine D2 partial agonist rather than a dopamine blocker, which is thought to cause few EPS. However, aripiprazole is a potent dopamine receptor blocker because it has low intrinsic activity for dopamine and higher affinity than the endogenous ligand dopamine. Therefore, aripiprazole may be at risk of inducing acute EPS in a dose-dependent manner (9). Epidemiologic studies quantifying this risk have shown that aripiprazole-induced EPS is not uncommon. In a nested case-control analysis, the odds ratio (OR) of EPS for aripiprazole was 5.38 (95% confidence interval [CI], 3.03–9.57). For dyskinesia, the risk for aripiprazole was 8.50 (95% CI, 8.53–2.27–31.97) compared to non-users (10). When aripiprazole-induced EPS appears in the masseter muscle, open bite may be observed. Given the assumed favorable side-effect profile of aripiprazole, its use in children and adolescents has increased. However, meta-analysis provides evidence for a non-negligible incidence of acute EPS in children and adolescents treated with aripiprazole (11). Therefore, adolescents taking aripiprazole may have developed open bite as a symptom of EPS.

## Conclusion

We present the first case of aripiprazole-induced open bite as a symptom of EPS. Antipsychotic-induced abnormal tension of masticatory muscles was assumed to be linked to the development of open bite. The incidence of EPS with aripiprazole is negligible in the literature, but it should be noted that this side effect is more likely to occur in adolescent patients. Open bite requires careful evaluation of the cause of malocclusion in order to provide comprehensive orthodontic treatment. Drug-induced open bite should always be considered as a cause that can be removed before orthodontic treatment.

## Data availability statement

The original contributions presented in the study are included in the article/supplementary material, further inquiries can be directed to the corresponding author.

## Author contributions

SS, RA, and KS were involved in the treatment of the presented cases, writing of the first draft, and editing the manuscript. TN and KO reviewed and edited the manuscript. AT and TN were a major contributor in writing the manuscript. All authors have read and approved the manuscript.

## Funding

This work was funded by JSPS KAKENHI Grant Numbers 22K10141.

## Conflict of interest

The authors declare that the research was conducted in the absence of any commercial or financial relationships that could be construed as a potential conflict of interest.

## Publisher's note

All claims expressed in this article are solely those of the authors and do not necessarily represent those of their affiliated organizations, or those of the publisher, the editors and the reviewers. Any product that may be evaluated in this article, or claim that may be made by its manufacturer, is not guaranteed or endorsed by the publisher.
